# Weight gain before and after switch from TDF to TAF in a U.S. cohort study

**DOI:** 10.1002/jia2.25702

**Published:** 2021-04-10

**Authors:** Patrick WG Mallon, Laurence Brunet, Ricky K Hsu, Jennifer S Fusco, Karam C Mounzer, Girish Prajapati, Andrew P Beyer, Michael B Wohlfeiler, Gregory P Fusco

**Affiliations:** ^1^ Centre for Experimental Pathogen Host Research School of Medicine University College Dublin Dublin Ireland; ^2^ St Vincent’s University Hospital Dublin Ireland; ^3^ Epividian Durham NC USA; ^4^ AIDS Healthcare Foundation New York NY USA; ^5^ NYU Langone Medical Center New York NY USA; ^6^ Philadelphia FIGHT Philadelphia PA USA; ^7^ Merck & Co., Inc Kenilworth NJ USA; ^8^ AIDS Healthcare Foundation Miami, FL USA

**Keywords:** antiretroviral therapy, Cohort, integrase strand transfer inhibitor, tenofovir disoproxil fumarate, tenofovir alafenamide, weight gain

## Abstract

**Introduction:**

Although weight gain has been reported with the use of integrase strand transfer inhibitors (InSTI), concurrent use of tenofovir alafenamide (TAF) has been implicated in recent studies. This study examined weight changes in people living with HIV (PLWH) who switched from tenofovir disoproxil fumarate (TDF) to TAF, to clarify the relative contribution to weight gain of core agents versus TDF to TAF switch.

**Methods:**

Antiretroviral‐experienced, virologically suppressed PLWH in the U.S. OPERA cohort were included if they switched from TDF to TAF (5NOV2015‐28FEB2019) and either maintained all other antiretrovirals or switched from a non‐InSTI to an InSTI. Linear mixed models were used to assess weight changes before/after the switch to TAF (restricted cubic splines on time) and rates of change over time (linear splines on time, based on the shape of the weight change curves). Changes in weight on TDF or TAF were assessed among those who maintained other antiretrovirals (overall, by core class), and those who maintained an InSTI or switched to an InSTI (by core agent). All models were adjusted for age, sex, race, (age‐sex, race‐sex interactions), BMI, CD4 cell count, endocrine disorders and concurrent medications that could affect weight.

**Results:**

A total of 6908 PLWH were included, with 5479 maintaining all other antiretrovirals (boosted protease inhibitor: 746, non‐nucleoside reverse transcriptase inhibitor: 1452, InSTI: 3281) and 1429 switching from a non‐InSTI to an InSTI (elvitegravir/cobicistat: 1120, dolutegravir: 174, bictegravir: 129). In adjusted models, modest weight gain was observed over time on TDF for most (0.24 to 0.71 kg/year); raltegravir was the exception with weight loss. Switching to TAF was associated with early, pronounced weight gain for all (1.80 to 4.47 kg/year). This effect with TAF switch was observed both in PLWH maintaining other antiretrovirals and those switching to an InSTI, regardless of which InSTI agent was used. Weight gain tended to slow down or plateau approximately nine months after switch to TAF.

**Conclusions:**

In this large, diverse U.S. cohort of PLWH, switching from TDF to TAF was associated with pronounced weight gain immediately after switch, regardless of the core class or core agent, suggesting an independent effect of TAF on weight gain.

## 1 INTRODUCTION

Switches from tenofovir disoproxil fumarate (TDF) to tenofovir alafenamide (TAF) have been common among people living with HIV (PLWH) since TAF was approved in the United States, often motivated by concerns around bone mineral density loss [1, 2, 3, 4, 5] and renal toxicity [6, 7, 8, 9] associated with TDF compared to TAF. However, there have been reports of increased weight gain with TAF use in antiretroviral therapy (ART)‐naïve PLWH in the ADVANCE trial [10, 11] and in a pooled analysis of eight clinical trials [12]. Similarly, weight gain has been reported with TAF use among virologically controlled ART‐experienced PLWH who switched to TAF from any other regimen [13] or from a TDF‐containing regimen [14, 15], and in PLWH who switched from TDF to TAF regardless of viral load [16].

Greater weight gain has also been reported with integrase strand transfer inhibitors (InSTI), compared to other core classes [11, 26], specifically with dolutegravir or bictegravir, compared to other agents [12, 30]. In the ADVANCE trial, weight increases were greater with dolutegravir+TDF/emtricitabine, compared to the South African standard of care of efavirenz+TDF/emtricitabine. It also showed that weight gain was greatest when TAF was used in conjunction with dolutegravir (dolutegravir+TAF/emtricitabine) [10, 11]. A U.S. cohort also reported greater weight increases with dolutegravir+TAF compared to other three drug regimens containing either emtricitabine or lamivudine (dolutegravir+TDF, dolutegravir+abacavir, elvitegravir/cobicistat/TAF, elvitegravir/cobicistat/TDF or other core+TDF) [31].

With growing concerns over the risks of weight gain with modern ART, the most recent U.S. HIV treatment guidelines summarize current evidence on weight gain with InSTIs or TAF, but note that “the clinical significance of these findings is still unknown” [32]. It is therefore essential to understand the respective roles of core agents versus TAF on weight gain. The objective of this study was to assess changes in weight before and after switching from TDF to TAF, among virologically suppressed PLWH in the United States, stratified by core agent.

## 2 METHODS

### 2.1 Study population

Data from the OPERA cohort, a database of electronic health records from 107,308 PLWH in care at 84 clinics across 18 U.S. states/territories, were utilized. ART‐experienced PLWH at least 18 years of age were included if they were virologically suppressed (last viral load <200 copies/mL) when they switched from TDF to TAF between 5NOV2015 and 28FEB2019. PLWH without a weight measurement within six months before or at any time after switch were excluded, as well as women with a pregnancy‐related diagnosis within six months before switch. Time on TDF was measured from the last of either initiation of the TDF‐containing regimen or 60 months prior to switch. After the switch from TDF to TAF, person‐time was censored at the first of TAF discontinuation, change in core agent, 12 months after the last clinical contact, death or study end (31AUG2019). Data for this analysis were collected between 5NOV2010 and 31AUG2019.

The OPERA database complies with all HIPAA and HITECH requirements, which expand upon the ethical principles detailed in the 1964 Declaration of Helsinki. The OPERA database received annual institutional review board (IRB) approval by Advarra IRB including a waiver of informed consent and authorization for use of protected health information.

### 2.2 Measurements

Weight and height were measured during routine clinical care, without a standardized protocol, and recorded in electronic health records. Repeated measures of weight in kilograms (kg) were employed. Endocrine disorders were defined as diabetes mellitus (type 1 or 2), hyperlipidaemia, hypothyroidism, hyperthyroidism, thyroiditis, hypogonadism and hypergonadism. Prescription of medications associated with weight gain within three months before/at switch were obtained from electronic health records. These consisted of antipsychotics and mood stabilizers, antidepressants, antihyperglycaemics, antihypertensives, oral corticosteroids, hormones, anticonvulsants, antihistamines, and appetite stimulants. Medications associated with weight loss within three months before/at switch included anti‐infectives, antineoplastics, bronchodilators, cardiovascular drugs, stimulants, antidepressants, antipsychotics, anticonvulsants, antihyperglycaemics, anti‐inflammatories, weight loss drugs and dementia treatment (Tables S1 and S2).

### 2.3 Analyses

Mean predicted weight was assessed over time before and after TDF‐to‐TAF switch. Analyses were performed among (1) PLWH who maintained all other agents (overall and by core class), (2) PLWH who maintained an InSTI (by core agent), and (3) PLWH who switched from a boosted protease inhibitor (PI) or non‐nucleoside reverse transcriptase inhibitor (NNRTI) to an InSTI (by post‐switch core agent). Multivariate linear mixed models were used to account for repeated measures. A random intercept was included to account for differences in weight at switch. Given the likely non‐linear trajectories of weight change before and after switch, time on TDF or TAF was modelled flexibly with restricted cubic splines. Knot placement was selected based on data distribution, with knots at −48, −12, 0 (time of switch), 3, 6, 12, 24 and 36 months. An interaction term between time and regimen was included to assess changes in weight over time in each regimen sub‐group.

The rate of change in weight (kg/year) was estimated over three time periods: (1) before switch, (2) from switch until nine months post‐switch, and (3) from nine months post‐switch until end of follow‐up, selected based on the shape of the curves. Multivariate linear mixed models with random intercepts were fit with linear splines on time (knots at zero and nine months). Statistical significance of rates of weight change was inferred from 95% confidence intervals (CI).

All models were adjusted for body mass index (BMI), age, sex, race, CD4 cell count, endocrine disorders and use of medications associated with either weight gain or weight loss, measured at the time of switch from TDF to TAF. BMI, age and CD4 cell count were measured continuously and centred at the mean. An interaction term between age and sex was included to control for the potential effect of menopause on weight. An interaction term between sex and race was also included to control for the association between ART and weight gain among women of colour [33].

## 3 RESULTS

### 3.1 Study population

A total of 6908 PLWH switched directly from TDF to TAF in the OPERA Cohort and met all inclusion criteria for this study. Among the 5479 PLWH who maintained other antiretrovirals (ARV), the most common core agents were darunavir for those on a boosted PI (68%), rilpivirine for those on an NNRTI (85%) and elvitegravir/cobicistat for those on an InSTI (73%, Figure S1A). Among the 1429 PLWH who switched from a non‐InSTI to an InSTI, the most common core agents co‐prescribed with TDF were darunavir or atazanavir for those on a boosted PI (42% and 44% respectively), and efavirenz for those on an NNRTI (84%, Figure S1B).

Demographic and clinical characteristics at the time of switch from TDF to TAF are described by core class for PLWH who maintained other ARVs, and overall for those who switched to an InSTI (Table 1). Groups were comparable for gender, ethnicity and weight‐related characteristics, although those who maintained a boosted PI and those who also switched to an InSTI tended to be older and were more likely to use medications associated with weight gain. The median duration of TDF use pre‐switch was 23.5 months (IQR: 13.1, 36.5). The median duration of follow‐up on TAF was 20.5 months (IQR: 10.7, 29.7); maximum follow‐up was 44 months.

**Table 1:** Demographic and clinical characteristics at TDF‐to‐TAF switch

	Maintained NNRTI, n = 1452	Maintained boosted PI, n = 746	Maintained InSTI, n = 3281	Switched to InSTI, n = 1429
Age, median (IQR)	45 (34, 54)	51 (42, 57)	44 (33, 52)	49 (39, 56)
Female, n (%)	274 (19)	154 (21)	494 (15)	252 (18)
Black, n (%)	589 (41)	292 (39)	1200 (37)	542 (38)
CD4 cell count, median (IQR)	717 (542, 940)	608 (441, 826)	654 (475, 867)	668 (493, 875)
Endocrine disorders^a^	270 (19)	188 (25)	651 (20)	322 (23)
Medications associated with weight gain^b^	404 (28)	275 (37)	988 (30)	485 (34)
Medications associated with weight loss^c^	267 (18)	169 (23)	649 (20)	273 (19)
Weight (kg), median (IQR)	81.6 (71.7, 93.4)	81.6 (70.8, 92.7)	80.7 (70.8, 92.5)	80.3 (70.8, 91.7)
BMI (kg/m^2^), median (IQR)	26.9 (24.0, 31.0)	27.1 (24.0, 30.7)	26.5 (23.8, 30.2)	26.6 (23.8, 30.1)
Underweight (BMI <18.5), n (%)	14 (1)	10 (1)	41 (1)	32 (2)
Normal weight (BMI ≥18.5 to <25), n (%)	463 (32)	239 (32)	1142 (35)	479 (34)
Overweight (BMI ≥25 to <30), n (%)	550 (38)	279 (37)	1240 (38)	554 (39)
Obese (BMI ≥30), n (%)	425 (29)	218 (29)	858 (26)	364 (26)

BMI, body mass index; InSTI, integrase strand transfer inhibitor; IQR, interquartile range; NNRTI, non‐nucleoside reverse transcriptase inhibitor; PI, protease inhibitor; TAF, tenofovir alafenamide; TDF, tenofovir disoproxil fumarate.

^a^ Type 1 Diabetes Mellitus, type 2 Diabetes Mellitus, hyperlipidaemia, hypothyroidism, hyperthyroidism, thyroiditis, hypogonadism, hypergonadism

^b^ Antipsychotics and mood stabilizers, antidepressants, antihyperglycaemics, antihypertensives, oral corticosteroids, hormones, anticonvulsants, antihistamines, or appetite stimulants

^c^ Anti‐infectives, antineoplastics, bronchodilators, cardiovascular drugs, stimulants, antidepressants, antipsychotics, anticonvulsants, antihyperglycaemics, anti‐inflammatories, weight loss drugs, dementia treatment.

### 3.2 Weight changes – maintained all other ARVs

**Figure 1:**
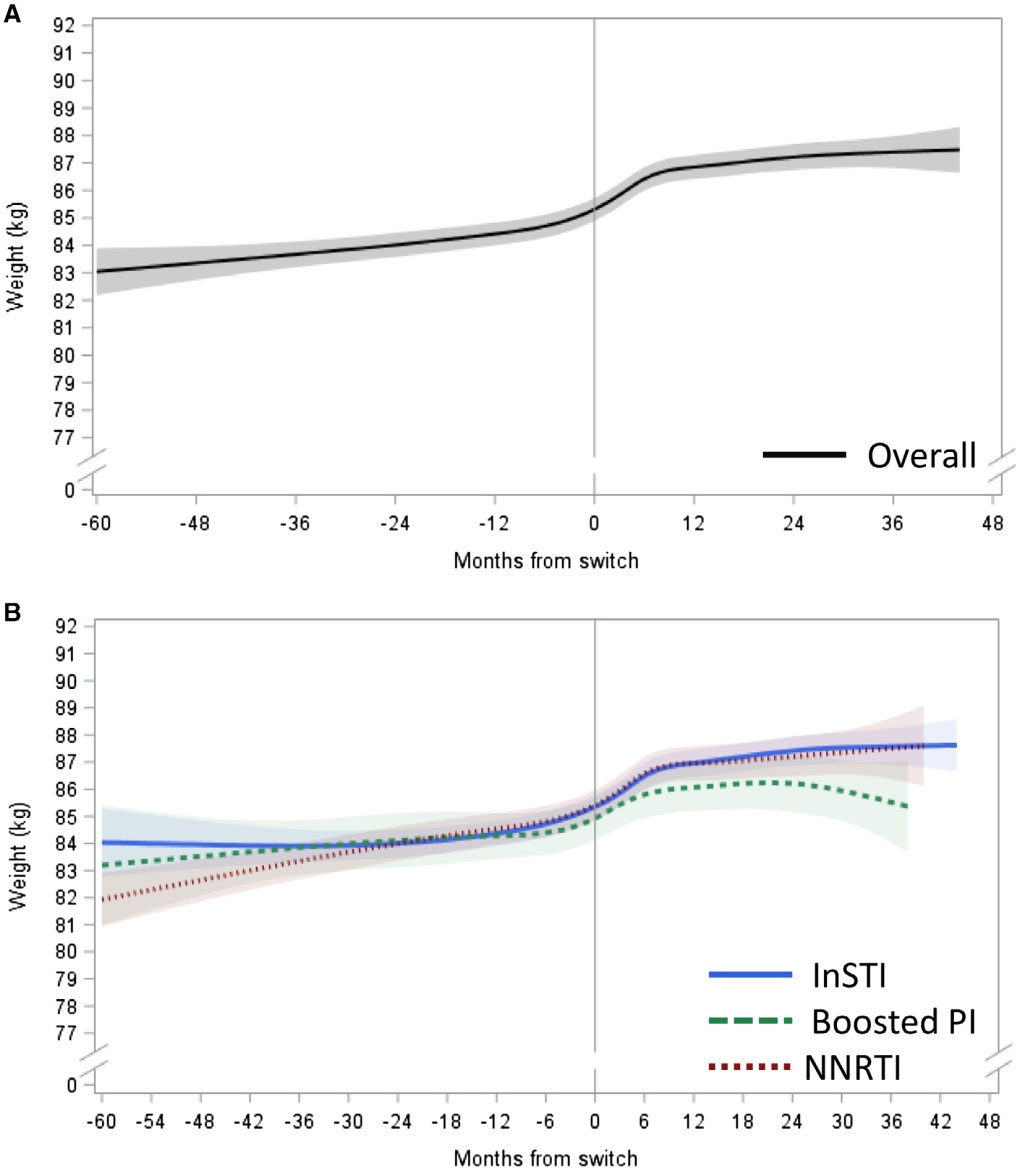
Adjusted predicted weight (kg) over time before and after TDF‐TAF switch among PLWH who maintained all other ARVs. **(A)** Overall, **(B)** by core agent class. Estimated with linear mixed model with restricted cubic splines on time; reference: 45 years old non‐Black man, BMI: 27, CD4 cell count: 700, no endocrine disorder, no medications associated with weight gain/loss. ARV, antiretroviral; BMI, body mass index; PI, protease inhibitor; InSTI, integrase strand transfer inhibitor; NNRTI, non‐nucleoside reverse transcriptase inhibitor; PLWH, people living with HIV; TAF, tenofovir alafenamide; TDF, tenofovir disoproxil fumarate.

Among PLWH who maintained all ARVs, the average adjusted rate of weight gain (kg/year) accelerated in the nine months following the switch from TDF to TAF. Beyond the first nine months of TAF, mean weights continued to increase at a slower rate, more comparable to the rate observed during treatment with TDF (Figure 1A). PLWH who maintained all other ARVs experienced average weight gains of 0.48 kg/year on TDF. Within the first nine months after switch, the average weight gain accelerated to 2.43 kg/year and then stabilized back to an average of 0.24 kg/year after the first nine months on TAF (Table 2). The average adjusted weight gained was 1.5 kg after 12 months and 1.9 kg after 24 months on TAF.

**Table 2:** Estimated rates of change in weight^a^ before and after switch from TDF to TAF

	On TDF 60 to 0 months	On TAF 0 to 9 months	On TAF 9+ months
	kg/year (95% CI)	kg/year (95% CI)	kg/year (95% CI)
Maintained all other ARVs			
Overall	0.48 (0.37, 0.59)	2.43 (2.15, 2.71)	0.24 (0.07, 0.41)
NNRTI	0.66 (0.51, 0.81)	2.25 (1.78, 2.71)	0.20 (−0.14, 0.54)
Boosted PI	0.31 (−0.02, 0.64)	1.98 (1.13, 2.83)	−0.11 (−0.57, 0.35)
InSTI	0.42 (0.26, 0.59)	2.64 (2.26, 3.01)	0.29 (0.08, 0.51)
Maintained an InSTI			
Elvitegravir/cobicistat	0.71 (0.53, 0.90)	2.51 (2.05, 2.96)	0.36 (0.12, 0.61)
Dolutegravir	0.73 (0.34, 1.11)	2.38 (1.64, 3.13)	−0.18 (−0.64, 0.28)
Raltegravir	−0.44 (−0.79, −0.08)	1.80 (0.57, 3.03)	0.63 (−0.20, 1.46)
Switched from non‐InSTI to InSTI			
Elvitegravir/cobicistat	0.24 (0.04, 0.43)	2.55 (1.86, 3.24)	0.26 (−0.10, 0.61)
Dolutegravir	0.22 (−0.08, 0.52)	3.09 (1.26, 4.93)	−0.23 (−1.62, 1.16)
Bictegravir^b^	0.01 (−0.38, 0.39)	4.47 (0.81, 8.13)	−9.97 (−23.79, 3.85)

ARV, antiretroviral; BMI, body mass index; CI, confidence interval; PI, boosted protease inhibitor; InSTI, integrase strand transfer inhibitor; NNRTI, non‐nucleoside reverse transcriptase inhibitor; TAF, tenofovir alafenamide; TDF, tenofovir disoproxil fumarate.

^a^ Estimated with linear mixed model with linear splines on time, adjusted for age, race, sex (interaction term between age‐sex and race‐sex), BMI, CD4 cell count, endocrine disorders and medications associated with weight gain/loss

^b^ limited follow‐up beyond nine months.

When stratified by class of core agent, PLWH who maintained an InSTI or an NNRTI experienced similar changes in weight over time (Figure 1B). Those on an InSTI gained on average 0.42 kg/year before switch, 2.64 kg/year within the first nine months after switch and 0.29 kg/year after nine months on TAF. Similarly, PLWH on an NNRTI experienced an average weight gain of 0.66 kg/year before switch and 2.25 kg/year within the first nine months after switch, but weight changes were not statistically significant beyond nine months on TAF (Table 2). A slightly different pattern was observed among PLWH who maintained the use of a boosted PI (Figure 1B), among whom the average rate of weight gain was not statistically significant while on TDF. On TAF, they gained on average 1.98 kg/year within the first nine months but experienced no statistically significant changes in weight beyond nine months on TAF (Table 2). The average adjusted weight gain at 12 months after switch among PLWH who maintained all other ARVs was 1.6 kg (InSTI), 1.1 kg (boosted PI) and 1.5 kg (NNRTI). At 24 months, the average adjusted weight gain was 2.1 kg (InSTI), 1.3 kg (boosted PI) and 1.5 kg (NNRTI).

### 3.3 Weight changes – maintained an InSTI

**Figure 2:**
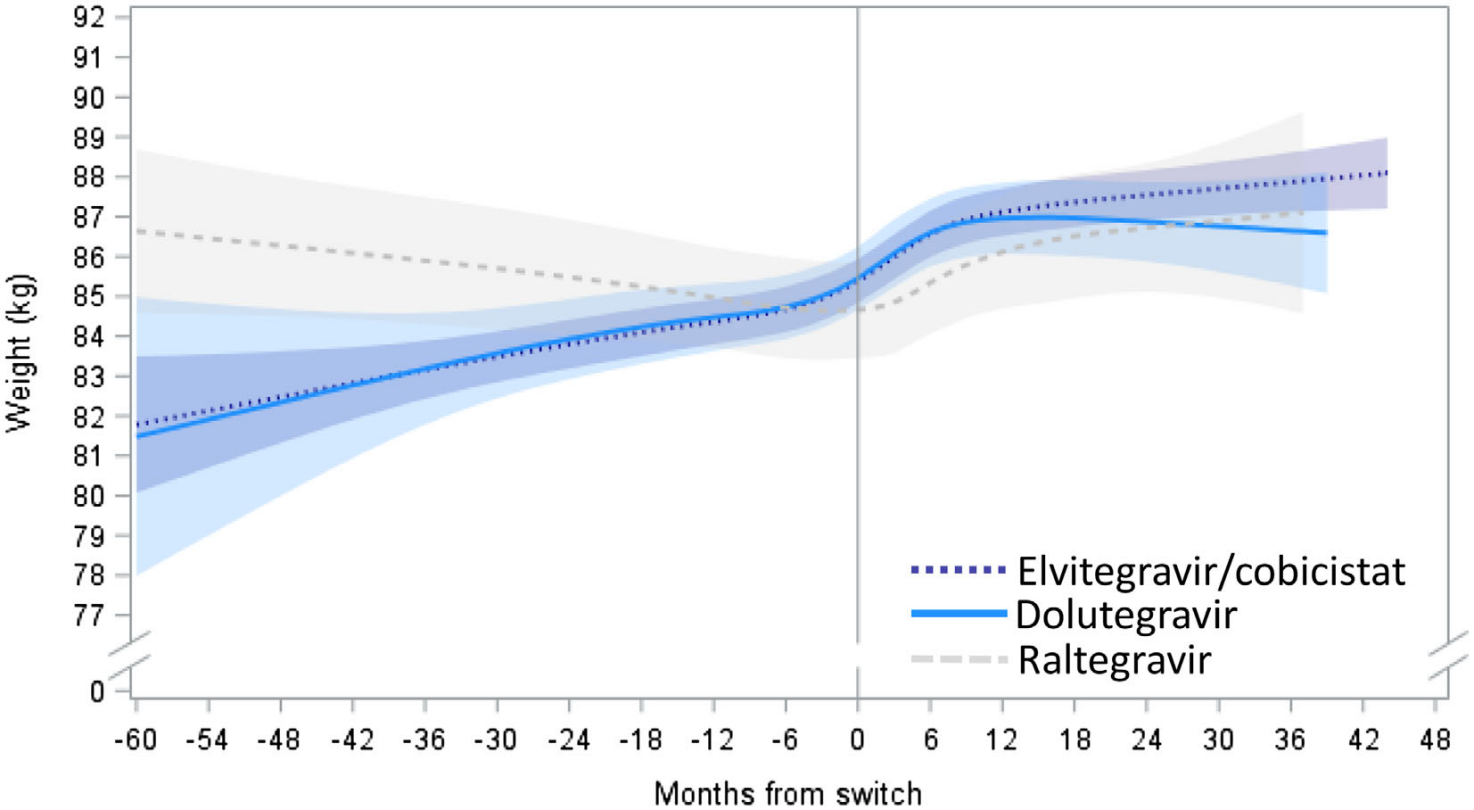
Adjusted predicted weight (kg) over time before and after TDF‐TAF switch among individuals who maintained an InSTI, by InSTI agent after switch. Estimated with linear mixed model with restricted cubic splines on time; reference: 45‐year‐old non‐Black man, BMI: 27, CD4 cell count: 700, no endocrine disorder, no medications associated with weight gain/loss. ARV, antiretroviral; BMI, body mass index; InSTI, integrase strand transfer inhibitor; PLWH, people living with HIV; TAF, tenofovir alafenamide; TDF, tenofovir disoproxil fumarate.

Among PLWH who maintained an InSTI, those on elvitegravir/cobicistat or dolutegravir experienced an increased rate of weight gain immediately following switch to TAF, followed either by lower rates of weight gain (elvitegravir/cobicistat) or a plateau (dolutegravir; Figure 2). For PLWH maintaining elvitegravir/cobicistat, the average rate of weight gain on TDF was 0.71 kg/year going up to 2.51 kg/year in the nine months immediately following switch to TAF, after which the average weight gain rate on TAF slowed down to 0.36 kg/year. As for those maintaining dolutegravir, the average rate of weight gain on TDF was 0.73 kg/year, going up to 2.38 kg/year in the first nine months after switch, followed by a plateau with no statistically significant weight gain over time after nine months on TAF (Table 2). PLWH on raltegravir experienced a slightly different pattern of weight changes over time (Figure 2). In contrast to elvitegravir/cobicistat and dolutegravir, an average weight loss of 0.44 kg/year was observed with raltegravir on TDF. Yet, similarly to the other InSTIs, an important, albeit less pronounced weight gain was observed with raltegravir immediately following the TDF‐to‐TAF switch, with average gains of 1.80 kg/year, followed by a plateau beyond nine months on TAF (Table 2). The average adjusted 12‐month weight gain since switch was 1.7 kg with elvitegravir/cobicistat, 1.5 kg with dolutegravir and 1.4 kg with raltegravir; the 24‐months average adjusted weight gain was 2.1 kg with elvitegravir/cobicistat, 1.4 kg with dolutegravir and 2.1 kg with raltegravir.

### 3.4 Weight changes – switched core agent

**Figure 3:**
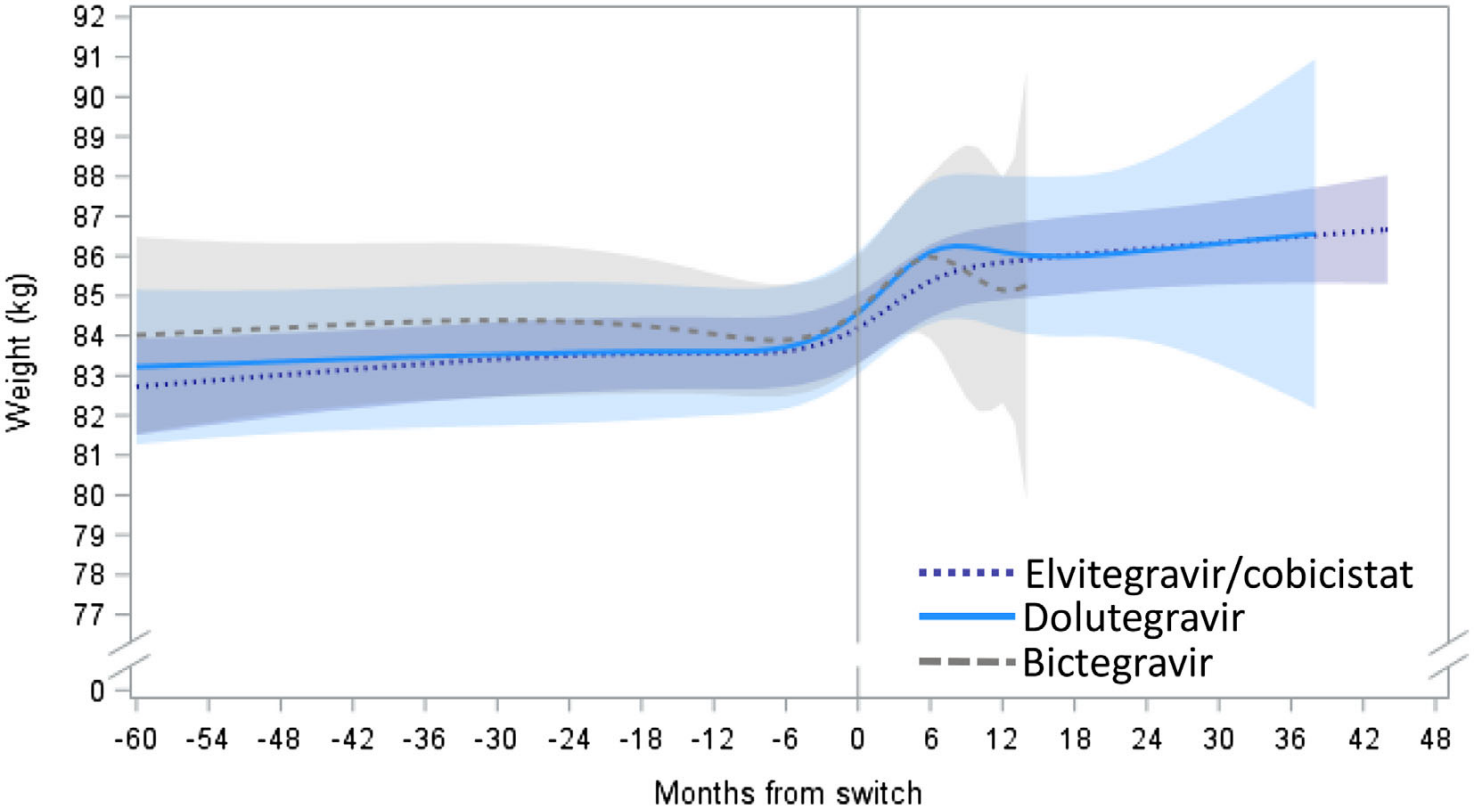
Adjusted predicted weight (kg) over time before and after TDF‐TAF switch among individuals who switched from a non‐InSTI to an InSTI, by InSTI agent after switch. Estimated with linear mixed model with restricted cubic splines on time; reference: 45‐year‐old non‐Black man, BMI: 27, CD4 cell count: 700, no endocrine disorder, no medications associated with weight gain/loss. ARV, antiretroviral; BMI, body mass index; InSTI, integrase strand transfer inhibitor; PLWH, people living with HIV; TAF, tenofovir alafenamide; TDF, tenofovir disoproxil fumarate.

Among PLWH who switched core agent class from a non‐InSTI to an InSTI at the time of switch from TDF to TAF, average adjusted weights were stable on TDF, followed by a sharp increase over the first nine months after switch and a return to stable weights after nine months of TAF (Figure 3). PLWH who switched to elvitegravir/cobicistat experienced weight gain rates of 0.24 kg/year on TDF, with an acceleration to 2.55 kg/year within the first nine months after switch. Beyond nine months of TAF and elvitegravir/cobicistat, no statistically significant changes in weight were observed. PLWH who switched to dolutegravir experienced weight gains at the rate of 3.09 kg/year within the first nine months after switch to TAF. However, no statistically significant changes in weight were observed on TDF or beyond nine months after switching to TAF and dolutegravir (Table 2). After 12 months of TAF, the average adjusted weight gains since switch were 1.6 kg with elvitegravir/cobicistat and 1.5 kg with dolutegravir. After 24 months, the average adjusted weight gains were 2.0 kg with elvitegravir/cobicistat and 1.6 kg with dolutegravir.

PLWH who switched to bictegravir experienced non‐significant rates of change in weight on TDF. Over the first nine months of TAF and bictegravir use, their average weight increased by 4.47 kg/year, beyond which they experienced no statistically significant changes in weight over time (Table 2). However, given the recent approval of bictegravir in the United States, data were insufficient to draw conclusions on patterns of weight gain with longer bictegravir exposures (Figure 3). Of note, only six PLWH switched to TAF and raltegravir, preventing modelling for this group.

## 4 DISCUSSION

This is among the first studies to comprehensively assess weight changes in a large and diverse population of PLWH switching from TDF to TAF in the United States. Weight gain concentrated within the first nine months after switch to TAF was consistently observed, regardless of the core agent used, with rates ranging from 1.80 to 4.47 kg/year in adjusted models. After this initial period of weight gain on TAF, weight increases slowed down to rates similar to those seen prior to switch. This dynamic weight gain was observed in both PLWH who maintained other ARVs and in PLWH who switched from a boosted PI or NNRTI to an InSTI, suggesting an independent effect of TAF on weight.

Several cohort studies, generally much smaller in size, have reported increases in weight after a switch from TDF to TAF. Only two studies modelled changes in weight both before and after a switch, each imposing an assumption of linearity on the data. In the Swiss HIV Cohort, the 2186 virologically suppressed PLWH who switched to dolutegravir experienced an average gain of 1.43 kg/year with TAF, and 0.70 kg/year without TAF; pre‐switch weight gain ranged from 0.33 to 0.48 kg/year [34]. In Italy, 252 PLWH who switched from rilpivirine/TDF/emtricitabine to rilpivirine/TAF/emtricitabine with a viral load <200 copies/ml had a stable mean weight before switch. Statistically significant gains from six months before switch to three months after switch were only observed among women (2.1kg) and those with a BMI >25 kg/m^2^ (1.6 kg) or a CD4 cell count ≤500 cells/μL (1.4kg) [15].

A study in Germany included a comparison group on TDF: over one year, the 129 PLWH who switched from TDF to TAF experienced a 3.17% mean increase in weight, whereas the 711 PLWH remaining on TDF experienced a 0.55% mean increase in weight [16]. Other studies did not estimate weight changes on TDF. In the United States, among 110 virologically suppressed PLWH maintaining all other ARVs, switching from TDF to TAF was associated with a 0.45 kg/m^2^ increase in BMI after six to twelve months on TAF (95% CI: 0.14, 0.76), with the pre‐switch BMI measured within a year before switch [14]. In Taiwan, 693 virologically suppressed PLWH who switched their current regimen to elvitegravir/cobicistat/emtricitabine/TAF experienced gains of 1.75 kg at 48 weeks, compared to gains of 0.54 kg in the 48 weeks prior to switch [13].

None of these studies reported a plateau in the rate of weight increases with TAF [13, 14, 15, 16, 34]. Follow‐up time on TAF was considerably shorter than in OPERA (six to eighteen months vs. fourty‐four months) and may have been too short to observe a plateau in the rates of weight gain after an initial rapid increase. Moreover, these studies did not model weight changes flexibly, and therefore lacked the ability to detect such a pattern. However, weight gain rates beyond nine months on TAF may have been underestimated in OPERA if the PLWH who gained the most weight discontinued their regimen earlier, thus not contributing to estimates for longer durations of TAF use.

These findings are adding to a mounting body of literature on the relationship between ART and weight gain. The marked increase in weight immediately following TAF initiation observed in OPERA was in line with results in ART‐naïve PLWH in clinical trials [10, 12] and in the CFAR Network of Integrated Clinical Systems (CNICS) cohort [31]. While multiple studies have shown greater weight gains with InSTI‐based regimen overall [11, 26] or specifically with dolutegravir, bictegravir or raltegravir [10, 11, 12, 35, 36, 37] compared to other regimens, most did not account for the role of TAF. In OPERA, the estimated rates of weight gain were numerically slightly higher in the first nine months of TAF when maintaining an InSTI, elvitegravir/cobicistat or dolutegravir, and when switching to bictegravir or dolutegravir, although differences between groups were not tested directly, and confidence intervals overlapped. Of note, elvitegravir was the most common agent among those who maintained an InSTI in OPERA and has been associated with the least weight gain within its class in trials; rilpivirine was the most common agent for NNRTI maintenance and has been associated with the most weight gain within its class [12]. However, the similarities in weight change observed across core groups do raise questions as to the role of concurrent switches to TAF in weight changes reported with switches to InSTI. Finally, the estimated weight loss over time on TDF observed in OPERA among PLWH who maintained raltegravir did not align with the literature suggesting weight gain with raltegravir even without TAF use [25, 26, 35, 36, 37]. However, this may reflect an age‐related decline in weight, as raltegravir was prescribed to an older population.

This study has several strengths. First, the study population was derived from the OPERA cohort, which includes a diverse population representing approximately 8% of PLWH in care in the United States and is representative of routine clinical care in the United States. Clinical diagnoses, prescriptions, and laboratory results are captured prospectively from electronic health records for all patients receiving healthcare at participating sites, thus providing complete and accurate clinical information reflecting real‐world clinical practices. In particular, the OPERA cohort provided a large study population of 6919 PLWH who switched from TDF to TAF that allowed for a novel but robust stratification of models by core agent class and specific InSTIs, providing unique insights into the role of specific regimens on weight gain with TAF. The longitudinal design utilized every single weight measurement to model changes in weight before and after switch. Linear mixed models with random intercepts were used to account for data correlation and differing weights at the time of switch. Restricted cubic splines on time were used to introduce flexibility and accurately represent changes in weight over time. By modelling weight over time on TDF, a baseline could be established for how much weight gain is expected without TAF. Therefore, the pronounced acceleration of weight gain observed immediately following switch can be interpreted in the context of weight changes that occurred over the five years prior to switch. Confounding bias was reduced through statistical adjustment, including endocrine disorders and the use of medications known to affect weight gain or weight loss. Interaction terms between age and sex as well as sex and race accounted for increased risks of weight gain after menopause and among women of colour [33]. In addition, by restricting the study to PLWH who were virologically suppressed at switch, bias arising from active viral replication and return to health was minimized.

However, this study is not without limitations. The absence of PLWH who maintained TDF use throughout the study period prevented direct comparisons between TDF and TAF. This concern was however alleviated by modelling weight changes on TDF before switch, as patterns of weight change on TDF before switch are likely representative of weight changes among PLWH who would have maintained TDF. Moreover, this study could not differentiate between the impact on weight of removing TDF versus adding TAF. Raltegravir use was not common, preventing its inclusion when assessing non‐InSTI to InSTI switches, and resulting in wide confidence intervals when assessing its maintenance. Similarly, bictegravir being only offered in combination with TAF, its maintenance could not be assessed. Because of its more recent approval, limited follow‐up time was available with bictegravir among PLWH who switched to an InSTI. Finally, residual confounding is possible. Notably, models were only adjusted for covariates measured at the time of switch, no adjustments were made for changes in viral load over time and data on adherence were not available. Moreover, marijuana use could not be controlled because the proportion of PLWH with documented marijuana use was too low and dietary habits are difficult to account for due to poor recall and limited documentation in electronic health records. While the potentially important role of sex and race in the relationship between ART and weight has been addressed through an interaction term, Black women constituted only 12% of the study population. CD4 cell counts and the specific core agents used prior to switching from TDF to TAF may also have had an impact on the results but were not controlled for in the analyses.

## 5 CONCLUSIONS

An early and pronounced weight gain was observed shortly after a switch from TDF to TAF, both in PLWH who maintained all other ARVs and in those who also switched to an InSTI‐based regimen, followed by a flattening of the curve after nine months of TAF use. Such patterns of weight gain were observed regardless of the specific InSTI agent used. These results, suggesting an independent effect of TAF on weight, are of clinical importance as increases in weight can impact long‐term clinical outcomes such as cardiovascular diseases, diabetes, fatty liver or other disorders. The potential long‐term clinical impact of the early, rapid, but transient increase in weight observed with TAF remains to be explored.

## Competing interests

PWGM’s institution has received research grants from GlaxoSmithKline, Gilead Sciences and Janssen Cilag; PWGM has received speaker honoraria from ViiV Healthcare, Gilead Sciences, Janssen Cilag, BMS. and MSD; and advisory board participation from ViiV Healthcare, Gilead Sciences, Janssen Cilag, BMS and MSD. LB, JSF and GPF are employees of Epividian, Inc. Epividian has had research funded by ViiV Healthcare, Merck & Co., Janssen Pharmaceutica and Gilead Sciences. RKH has received research grants from Gilead Sciences and Janssen, speaker honoraria and advisory boards from ViiV Healthcare, BMS, Merck, Gilead Sciences and Janssen, and advisory board participation with ViiV, Gilead Sciences, Janssen, and Epividian. KCM has received research grants from Gilead Sciences, Merck, Janssen, and GSK/ViiV Healthcare and honoraria for Speakers Bureau and Advisory Boards from Gilead Sciences, Merck, Janssen and GSK/ViiV Healthcare; and advisory board participation with Epividian. APB and GP are employees of Merck Sharp & Dohme Corp., a subsidiary of Merck & Co., Inc, Kenilworth, NJ, USA. MBW has participated in post‐conference advisory boards for the Conference on Retroviruses and Opportunistic Infections (CROI) and International AIDS Conference (IAC) and also serves as a principal investigator on ViiV Healthcare clinical trials but does not receive personal compensation for this work, which goes directly to the AIDS Healthcare Foundation.

## Authors’ contributions

LB, PWGM, JSF, GP and APB share the responsibility for the design of this study. LB conducted all the analyses. PWGM, LB, RKH, JSF, KCM, GP, APB, MBW and GPF contributed to the interpretation of results. LB drafted the manuscript. All authors have critically reviewed and approved the manuscript and have participated sufficiently in the work to take public responsibility for its content.

## Abbreviations

ART, antiretroviral therapy; ARV, antiretroviral; BMI, body mass index; c, cobicistat; CKD, chronic kidney disease; InSTI, integrase strand transfer inhibitors; IQR, inter‐quartile range; IRB, institutional review board; NNRTI, non‐nucleoside reverse transcriptase inhibitor; PI, protease inhibitor; PLWH, people living with HIV; TAF, tenofovir alafenamide; TDF, tenofovir disoproxil fumarate; U.S., United States.

## Funding

This work was supported by Merck Sharp & Dohme Corp., a subsidiary of Merck & Co., Inc., Kenilworth, NJ, USA.

## Supporting information


**Table S1**. Medications associated with weight gain
**Table S2**. Medications associated with weight loss
**Figure S1**. Core agents before and after TDF‐to‐TAF switch.
