# Supplementary material for: Weight gain before and after switch from TDF to TAF in a U.S. cohort study
Source: J Int AIDS Soc. 2021 Apr 10;24(4):e25702. doi: 10.1002/jia2.25702 (PMC8035674; doi:10.1002/jia2.25702)
Supplement: Supplementary file 1 — Table S1. Medications associated with weight gain Table S2. Medications associated with weight loss Figure S1. Core agents before and after TDF‐to‐TAF switch. [file JIA2-24-e25702-s001.docx]

**Supplemental Table 1.** Medications associated with weight gain

| Category | Drugs |
| --- | --- |
| antipsychotics and mood stabilizers | chlorpromazine  clozapine  haloperidol  lithium  olanzapine  valproic acid  risperidone  quetiapine  fluphenazine |
| antidepressants | phenelzine  tranylcypromine  citalopram  amitriptyline  nortriptyline  mirtazapine  paroxetine  escitalopram  fluvoxamine  sertraline |
| antihyperglycemics | insulin  chlorpropamide  gliclazide  glipizide  glyburide  pioglitazone  rosiglitazone  repaglinide  tolbutamide  glimepiride/rosiglitazone  alogliptin/metformin  alogliptin/pioglitazone  canagliflozin/metformin  dapagliflozin/metformin  empagliflozin/linagliptin  empagliflozin/metformin  glimepiride/pioglitazone |
| antihypertensives | atenolol  metorprolol  nadolol  penbutolol  pindolol  propranolol  sotalol  betaxolol  bisoprolol  bisoprolol/hydrochlorothiazide  hydrochlorothiazide/metoprolol  nebivolol |
| oral corticosteroids | cortisone  prednisolone  prednisone  methylprednisolone  hydrocortisone |
| hormones | human growth hormone/somatropin  medroxyprogesterone  ulipristal |
| anticonvulsants | gabapentin  valproic acid/sodium valproate  carbamazepine |
| antihistamines | cyproheptadine  diphenhydramine |
| appetite stimulants | megestrol  oxandrolone  dronabinol  marinol  tetrahydrocannabinol |

**Supplemental Table 2.** Medications associated with weight loss

| Category | Drugs |
| --- | --- |
| anti-infectives | metronidazole  amphotericin  atovaquone  pyrimethamine  ethionamide |
| antineoplastics | aldesleukin and interleukin-2  capecitabine  carboplatin  cytarabine  dacarbazine  fluorouracil  hydroxyurea  imatinib  irinotecan  methotrexate  vinblastine sulphate  vinorelbine tartrate |
| bronchodilators | salbutamol sulphate  theophylline |
| cardiovascular drugs | amiodarone  acetazolamide  hydralazine HCl  quinidine |
| stimulants | methylphenidate HCl  phentermine  amphetamine  dextroamphetamine  methamphetamine  pemoline  dexmethylphenidate  phendimetrazine  atomoxetine  modafinil  armodafinil |
| antidepressants | fluoxetine  bupropion |
| antipsychotics | loxapine |
| anticonvulsants | topiramate |
| antihyperglycemics | exenatide  liraglutide  semaglutide  dulaglutide |
| anti-inflammatories | sulphasalazine |
| weight loss drugs | bupropion-naltrexone  liraglutide  lorcaserin  orlistat  phentermine-topiramate  diethylpropion  phentermine |
| dementia treatment | galantamine  rivastigmine |

**
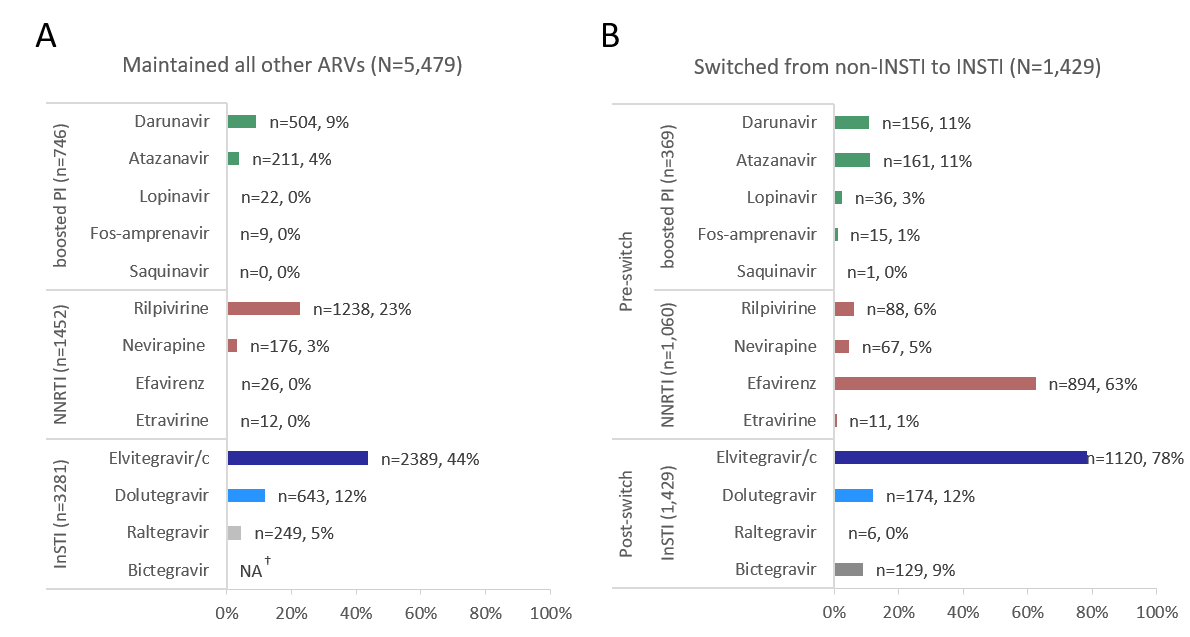
Supplemental Figure 1. Core agents before and after TDF-to-TAF switch.** (A) Individuals who maintained all other ARVs (N=5,479), (B) Individuals who switched from a non-InSTI to an InSTI (N=1,429); percentages out of the overall study populations.

ARV, antiretroviral; PI, protease inhibitor; InSTI, integrase strand transfer inhibitor; N, number; NA, not applicable; NNRTI, non-nucleoside reverse transcriptase inhibitor

^†^ Bictegravir is only available in coformulation with TAF and emtricitabine.
